# A real-world data challenge: guidance for aligning data privacy compliance and fit-for-purpose usability

**DOI:** 10.1093/haschl/qxaf210

**Published:** 2025-11-12

**Authors:** Dena H Jaffe, Bradley A Malin, Rachele M Hendricks-Sturrup

**Affiliations:** Oracle Health, Petah Tikva 4951448, Israel; Department of Biomedical Informatics, Vanderbilt University Medical Center, Nashville, TN37203, United States; Duke-Robert J. Margolis, MD, Institute for Health Policy, Duke University, Washington, DC 20004, United States

**Keywords:** real-world data, RWD, EHR, HIPAA, GDPR, de-identification, anonymization

## Abstract

The growing use of real-world data (RWD), particularly from electronic health records (EHRs), has heightened the need for careful attention to data privacy, utility, and transparency. We examine the complex processes involved in curating privacy-compliant EHR-derived RWD, highlighting key de-identification considerations and techniques. We emphasize the importance of aligning curation practices with privacy laws and regulations, with a particular focus on the comprehensive documentation of de-identification techniques. Such documentation should reflect intended data use, accessibility, availability, accuracy, and granularity. For researchers, greater transparency in these practices can improve compliance and lead to more robust and reliable real-world evidence. For policymakers, it provides a foundation to develop more specific and actionable guidelines and oversight mechanisms. Ultimately, highly transparent curation process enhances the reliability of RWD and supports rigorous, nuanced, and informed decision-making across the health care ecosystem.

## Teaser

In today's data-driven health care ecosystem, the promise of real-world evidence hinges on the quality, transparency, and privacy compliance of the real-world data we curate—especially from electronic health records (EHRs). This article unpacks the operational and regulatory trade-offs inherent in transforming messy, heterogeneous EHR data into reliable, fit-for-use datasets. By examining de-identification techniques, privacy risks, and curation practices, we argue that comprehensive, transparent documentation is not just a compliance exercise but the key to unlocking trustworthy, high-quality evidence. For researchers, this transparency drives methodological rigor; for policymakers, it lays the foundation for more specific, actionable oversight. Our conclusions are clear: only through careful documentation, privacy-first practices, and fit-for-purpose data management can we ensure that the real-world data fueling tomorrow's evidence is valid, reliable, and ethically sound.

## Introduction

The use of electronic health records (EHRs) to store patient data digitally has revolutionized health care management and biomedical research. The contribution of EHRs and other sources of real-world data (RWD) as real-world evidence (RWE) for regulatory submissions and policy decision-making is rapidly proliferating and accelerating research within and outside of biomedicine and medical practice.^1-3^ In contrast to controlled clinical trial designs, RWD-driven research offers an opportunity to produce timely insights into routine clinical care and practice, the examination of unintended or rare outcomes, and the inclusion of larger and more diverse patient populations. Similar to clinical trials, generating RWD as a source of evidence requires adherence to rigorous standards that include anonymization and transparency to broadly protect patient privacy and ensure valid outcomes.^1,4,5^ Over the past decade, a multilayered system of concepts and guidance has evolved to evaluate and determine what constitutes fit-for-use RWD.^1,2,4,6^

Curation processes for RWD are frequently performed entirely or jointly by data owners, health data software suppliers, commercial health data analytics providers, and research and public health surveillance networks. Compliance with privacy requirements may occur prior to or as part of the data management processes and is often contingent on contractually binding data use agreements.

Despite extensive literature and guidance on producing fit-for-use data and ensuring privacy compliance, these topics are typically addressed independently.^5-12^ What has resulted is siloed thinking in practice and a dearth of essential thought leadership and guidance at the intersection of these topics. This article aims to address this issue. Here, we offer unique guidance for those who seek to offer transparency around ensuring data privacy and utility at the RWD curation phase to generate fit-for-use RWE.^13^ Researchers should have an underlying understanding of the operational impact of relevant privacy laws and regulations, which will give policymakers further context needed to align and exact guidelines and policies on EHR and other RWD management. Therefore, we also provide such insights and discuss patient privacy guidance and compliance standards for researchers within and outside of the United States, implementation factors to consider when ensuring privacy protections for patient-level RWD, and de-identification techniques used to protect patient privacy. Last, we describe considerations to produce fit-for-use RWD derived from EHRs, relevance, reliability, impacts of privacy compliance, and transparency of this process.

### Patient privacy guidance and compliance standards for research

When using data for secondary purposes beyond primary care, patient privacy compliance focuses on risk mitigation of patient uniqueness.^10^ Here we demonstrate this as an international challenge by examining 2 major centers of use—the United States and Europe. In the United States, health data generated by health care providers and insurers are regulated by the Health Insurance Portability and Accountability Act (HIPAA), whereas in the European Union privacy in the context of secondary data use is covered by the General Data Protection Regulation (GDPR).^14-16^ As part of health care, patient health records that include personally identifiable information (PII), including protected health information (PHI), are generally inaccessible from sharing or using outside of direct patient care without additional oversights such as internal review boards or data use agreements.^15,16^ However, use for healthcare research, which is beyond the original intent of the data collected, is permissible when data are de-identified (HIPAA), anonymized (GDPR), or pseudonymized (GDPR). According to HIPAA, which regulates PHI, de-identification is the removal of sufficient information such that the data “neither identifies nor provides a reasonable basis to identify an individual.”^16,17^ Under GDPR, that refers to personal data, anonymization is the process of irreversibly altering personal data so that persons are no longer identifiable by “all the means reasonably likely to be used.”^16^ Both HIPAA and GDPR note that upon de-identification or anonymization, the laws protecting patient privacy no longer apply at the federal level; this does not, however, preempt stricter US state or European member state-specific privacy laws.^14,18,19^

In the United States, the HIPAA Privacy Rule provides 2 implementation methods to achieve the definition of de-identified: Safe Harbor (SH) and Expert Determination (ED).^15,17^ Safe Harbor requires both the removal of 18 specific identifiers from the health data and having no actual knowledge of residual information for identifying individuals.^17,20^ This formulaic approach is limited by inflexible controls, reduced data utility, and varying interpretations. Alternatively, the principle-based ED requires “a person with appropriate knowledge of and experience with generally accepted statistical and scientific principles and methods for rendering information not individually identifiable.”^17^ Researchers can benefit from the adaptability of this methodology that considers their priorities when designing a HIPAA-compliant dataset, although with the drawbacks of potential increased time and financial costs, the inherent subjectivity of the evaluation, and the limited understanding of the process by the data end-user. In Europe, GDPR aims to harmonize privacy and data protection standards across member states but does not explicitly prescribe de-identification techniques required to achieve patient anonymization.^16^ Member states implementing GDPR often incorporate additional considerations.^19,21^ Like ED in the United States, GDPR relies on expert judgement and documentation of anonymization or pseudonymization to align with regulatory requirements.^22^

## Methodology for assessing risk for patient privacy protection for RWD

Regulatory conformance with patient privacy rules considers risk threshold levels for re-identification. While cutoff points are not explicitly formulated by HIPAA or GDPR, several regulatory agencies, notably the European Medicines Agency's Policy 0700 and Health Canada's Public Release of Clinical Information (PRCI) initiative have defined a re-identification threshold of 0.09 (1 in 11 patients) for clinical trial reports intended for public release.^23,24^

At its core, re-identification risks relate primarily to patient data at risk of disclosure and there are many ways to mitigate such risks. In the United States in particular, a comprehensive approach to privacy protection and the resultant data amendments also considers the data environment and users, often referred to as the “onion-skin” principle (Table 1).^12,20,25^ The environment reflects the accessibility and technical security measures surrounding the data that limit access by adversaries to breach sensitive information. It is characterized as a controlled or closed-access, registered or limited-access, or publicly accessible system. At this level, technical barriers can be implemented to mitigate the likelihood of malicious attacks, such as secured access environments and/or a governance system with clearly defined operational protocols, which function to protect against privacy breaches. For instance, from a technical perspective, data could be sandboxed in a virtualized enclave environment that prohibits users from downloading the data. Additionally, the manager of the sandbox could apply fine-grained access logging to ensure an audit trail can be produced and assessed, whereas, from a nontechnical perspective, data users could be required to submit requests for access to a data access committee and/or enter into contractually binding data use agreements that prohibit the linkage of the data with other resources, as well as any re-identification attempts on the data. The final and most recognized layer of privacy protection involves data amendments applied to specific fields, codes, and values. Processing and curating these data require rigorous review and ongoing maintenance concerning their classification as either direct personal or patient health identifiers or as indirect or quasi-identifiers that single a patient out.

**Table 1.** Privacy protection considerations for fit-for-use data derived from electronic health records.

Environment	User type	Privacy-related data amendments
Public	Public	High
Registered	Data-sharing agreement	Moderate
Controlled	Covered entity/business associate Data-sharing agreement Governance/data review committee	Low–moderate

As opposed to direct identifiers, the intrinsic value of quasi-identifiers resides in the relationship they have with other fields in the dataset and with publicly available data. They are considered to pose moderate risks of re-identification, and for HIPAA ED and GDPR, require context and consideration. Table 2 presents some of the core quasi-identifying attribute groups examined for the de-identification of RWD, including demographic characteristics, social determinants of health (SDOH), geography, lifestyle characteristics, public data, and publicly recorded events. Many of these data elements are critical when creating control arms for clinical trials, designing target trials, or conducting pharmacoepidemiology studies.^31,32^ For example, incorporating more detailed demographic variables in these studies helps refine patient inclusion criteria, approximates randomization, controls for confounding, and enhances generalizability.

**Table 2.** Core quasi-identifying attribute groupings.

Risk	Examples^a^
Demographic characteristics	Age
	Country of origin or residence
	Ethnicity
	Marital status
	Number of children
	Race
	Religion
	Sex/gender identity
	Spoken language
Social determinants of health	Education
	Employment
	Income
	Occupation
	Social determinants of health indices
Geography	City
	State
	Street address
	Census tract
	Postal code
	Urban/rural
Lifestyle characteristics	Countries visited
Publicly recorded events	Age-related events (eg, newborns, elderly)
	Assault or homicide
	Birth and death dates
	Extreme health status (eg, morbidly obese)
	Legal intervention
	Military, war, or terrorism
	Vehicle accident
	Weightlessness

^a^Sources of quasi-indicators may be linked to dataset from nationally collected and publicly available datasets in the United States include the American Housing Survey (AHS),^26^ Healthcare Cost and Utilization (HCUP) datasets,^27^ Social Determinants of Health (SDOH) indices databases,^28^ the National Health and Nutrition Examination Survey (NHANES),^29^ and Rural-Urban Commuting Area Codes (RUCA).^30^

Risk, therefore, is a combination of the applications that reduces the chance that an attack is committed and the chance that the attack is successful given that the attack is committed. In general, public environments and broader user access require greater levels of data modifications, while restricted environments and user access require fewer amendments (Table 1). For example, data in a closed environment with a low chance of attack (eg, 0.5) and where patient uniqueness is low (eg, 0.18) meet the minimum threshold level of protection from re-identification (0.5 × 0.18 = 0.09). However, these data in the public domain without oversight have a high risk of attack (eg, 1) and, therefore, exceed the commonly accepted minimal protection level (1 × 0.18 = 0.18).

Importantly, particularly for ED- and GDPR-compliant datasets, privacy compliance is dataset specific. When datasets are linked, they require further risk assessment due to the new set of data combinations and risk strata.

### De-identification techniques

Over the past decade, there has been a proliferation of de-identification techniques applied to direct and quasi-identifiers.^9,11,33-38^ Table 3 presents 6 de-identification techniques: (1) tracking—a privacy-preserving record linkage technique that preserves the ability to follow patients longitudinally and linked across datasets; (2) masking—the obscuring of specific identifiers with techniques such as partial redaction, substitution, and blurring that can be applied to texts, dates, and binary large object (BLOB) data types (eg, images and voice data); (3) generalization—the aggregation or top coding to reduce the granularity or specificity of the data while maintaining some value; (4) redaction—the removal of data fields or values by suppressing or filtering the text, numeric, or date type data; (5) disruption—the modification of data to maintain the characteristics of the dataset by adding noise or rearranging individual-level values; and (6) randomization—the substitution or abridging of data in order to create imputed data or images.

**Table 3.** Privacy methods: data de-identification techniques.

De-identification techniques	Description	Data types^a^	Examples		
Tracking^9,11,33^	Privacy-preserving ability to follow patients				
Pseudonymization Tokenization Encryption Hashing Obfuscation Coding	Replace data with a surrogate value of no intrinsic value or related to the original value. Original data may be retrieved using a mapping table or key; uni- or bidirectional or random transformation using cryptographic methods	Text and numeric Patient ID SSN Device number	Social Security number: 123-45-6789 → gAB91K13d533		
Masking^9,11,33-37^	Modify data by obscuring				
Partial redaction Character replacement	Hide part of a data field or person	Text, numeric, date, and time Patient ID SSN Birth date	Social Security number: 123-45-6789 → XXX-XX-6789 Birthdate: “09-Nov-1980” → “1980”		
Data substitution Randomization	Replace real values with fictitious alternatives	Text, numeric, date, and time Patient names Places Brand names	Patient name: “John Doe” → “James Smith”		
Date shifting (substitution)	A specific subset of data substitution whereby dates are modified by adding or subtracting a random number of days per patient, while preserving the relative interval between dates	Date and time Birth date Encounter date Medication dispensing date	Original data		
			Patient ID	Admission date	Discharge date
			001	2023-01-01	2023-01-10
			002	2023-02-15	2023-02-20
			003	2023-03-01	2023-03-02
			Date-shifted data		
			Patient ID	Admission date	Discharge date
			001	2023-01-06	2023-01-15
			002	2023-02-08	2023-02-13
			003	2023-02-21	2023-03-22
Blurring Pixelation Distortion Mask-out	Modifying the image by altering visual elements	Binary	3 × 3-Pixel block in grayscale blurring with averaging of neighbors		
			Original:		
			120	125	130
			118	122	135
			110	115	128
			Blurred:		
			121	124	127
			120	123	126
			117	120	123
Extraction and replacement deep learning (DL)	Deep learning–based techniques for substituting speaker identity features only	Binary	Audio → speaker extraction → replacement (speaker_original to speaker_anonymized) → synthesis → evaluation		
Signal modification	Signal processing is used to modify speech signals	Binary	Apply a warping function compressing all frequencies >500 Hz by 20%		
Generalization^9,11,33^	Reduce the granularity of the data while maintaining some value				
Aggregation Rounding	Group values into broader categories	Text and numeric Race Marital status Age	Age:		
			Patient ID	Age (years)	Age (years) aggregated
			001	14	10-14
			002	47	45-49
			003	82	80-84
Top or bottom coding	Group specific values into broader categories	Numeric Age BMI Income	Age:		
			Patient ID	Age (years)	Age (years) top coded
			001	14	14
			002	92	89
			003	89	89
Redaction^9,11,33^	Remove data fields or values				
Suppression Filtering	Replace values with null values or exclude data fields from dataset	Text, numeric, date and time Name Address Ethnicity Cause of death	Exclude patient address field Condition code: “R99”^b^ → “NULL”		
Disruption^9,11,33^	Modify data to maintain the characteristics of the dataset				
Noise addition Perturbation	Modify individual data points with random or controlled variation while preserving overall trends and aggregate statistics	Numeric Salary Age BMI	Original data:		
			Patient ID	Income ($)	
			001	50 000	
			002	60 000	
			003	45 000	
			Random noise within ±5% of original:		
			Patient ID	Income ($) + noise	
			001	51 500	
			002	63 000	
			003	43 650	
Data shuffling	Rearrange values of a particular field preserving the exact values but removing the relationship with the individual	Text and numeric Geography Diagnosis Income	Original data:		
			Patient ID	Age	State
			001	45	Ohio
			002	36	Florida
			003	82	Texas
			Shuffled data:		
			Patient ID	Age	State
			001	45	Texas
			002	36	Ohio
			003	82	Florida
Randomization^9,11,34^	Substituting or abstracting information across the entire dataset				
Imputed data	Replace the original entirely while retaining patterns and distributions	All data types	Original data:		
			Patient ID	Age	HbA1c (%)
			001	45	4.1
			002	37	5.3
			003	82	7.2
			Synthetic data:		
			Patient ID	Age	HbA1c (%)
			001	35	5.4
			002	48	4.0
			003	81	7.1
Synthetic images Face shifting	Use of Generative Adversarial Networks (GANs) to generate synthetic images	Binary	Original face is replaced with GAN model–generated face while keeping rest of photo contextually intact		
Subsampling	Sample of the original dataset	All data types	Random sample of 5%-10% of dataset		

Abbreviations: BLOB, binary large object; BMI, body mass index; HbA1c, glycated hemoglobin; ID, identification number; SSN, Social Security number.

^a^Data types include text, numeric, date and time, binary (eg, BLOBs such as audio and video files, images, and PDFs), structured (eg, array, list), semi-structured (eg, geospatial, JSON, XML), unstructured (eg, notes, emails), and biometric data.

^b^Condition code R99 is an International Classification of Diseases (ICD) version 10 code for ill-defined and unknown cause of mortality.

### Fit-for-purpose data management and usability

The production of fit-for-purpose RWD sourced from EHRs for RWE requires comprehensive data management to ensure that the data are relevant, reliable, and of high-quality for their intended and appropriate use. Recent tools guiding and ensuring fit-for-purpose uses of structured EHR data in clinical research and for RWE include, but are not limited to, the SUITABILITY Framework by the International Society for Pharmacoeconomics and Outcomes Research (ISPOR) and the Fit-for-Purpose Assessment by the US Food and Drug Administration (FDA) (**Table S1**).^5-7,39^ The management of RWD is typically the responsibility of data custodians who oversee the data curation processes to ensure the implementation of quality controls and assessments, proper documentation, and alignment with intended database purposes.

Within their respective frameworks, both ISPOR and the FDA emphasize the importance of applying privacy compliance techniques to source data. The ISPOR SUITABILITY Framework identifies data privacy as an important component of data governance, stating the need to “provide evidence that appropriate national law and regulations for secondary use of the EHR-derived data were observed.”^5^ Indirectly, this framework alludes to the inclusion of privacy-related data transformations as part of the guidance for documenting data provenance, specifically, “that tracks a piece of data from its origin to where it is presently.”^5^ ISPOR highlights the protection of patient health data in RWD as a fundamental requirement for federated data sharing. It also considers privacy to be a significant factor when implementing FAIR (fair, accessible, interoperable, and reusable) principles and artificial intelligence in health care.^40^ Similarly, the FDA's Fit-for-Purpose Assessment underscores the patient's privacy protection as a core consideration for data linkage efforts, requiring documentation of “any data transformations, including any modifications made for privacy protection.”^17^

## Relevance and reliability and the impact on privacy compliance

The application of de-identification techniques for health data privacy compliance varies according to the underlying regulatory framework. Under the HIPAA SH method, typical techniques include redaction of patient identifiers, masking of dates to year (or date shifting), generalization of geographies, and top-coding of older ages.^37^ In contrast, HIPAA ED and GDPR (Recital 26) assess context and risk and do not explicitly recognize any 1 technique.^16,17^

We recommend that researchers reflect and document the lineage and consequences of de-identification techniques on RWD, which are foundational to transparency and HIPAA privacy.^8^ The following are important considerations in the design and curation of fit-for-use RWD sourced from EHRs, which drive the type and extent of amendments applied.

Data curation starts by defining the intended use of the RWD. For example, a general-purpose EHR dataset seeks to provide analytic data that maintain a high degree of availability of patient characteristics, true and complete dates, and population representativeness, which, as noted above, are important contextual variables in real-world studies. Resultant techniques may partially redact dates of death to month and year, aggregate ethnicity into 2 groupings, and suppress unique or public events, such as military-related diagnoses, admissions, and discharge codes. Of obvious consequence is the limited relevance to studies with populations and outcomes that are included as quasi-identifiers. Knowledge of the dataset's purpose (ie, relevance) is essential for understanding potential bias (eg, selection bias, information bias) and generalizability to the target population.

Next, curation balances privacy compliance using HIPAA's ED method and GDPR guidance that is predicated on a low risk of data breach and dependent on the environment and users’ accessibility to relevant data. For example, the All of Us Research Program relies upon a tiered data access model with increased data availability and granularity for their registered vs controlled tier data.^41^ Other large, population-based, aggregated datasets live in controlled environments with restricted access to data providers with data use agreements.^42^ These closed environments give data aggregators and users broader, more relevant data to address widely applicable research questions. Federated data models, such as the Observational Medical Outcomes Partnership (OMOP) and Patient-Centered Outcomes Research Institute PCORnet data models, in quasi-public data environments place the responsibility of maintaining data privacy compliance with the data holder.^43,44^

Amending data to minimize identifiability most often includes and affects data timeliness or the temporality or period of data availability. Date-shifting or suppression are de-identification techniques that change data availability period.^37^ Date shifting substitutes a patient's dates with a random number of days and preserves the relative interval between dates. When shifting is additive (eg, between +1 and +365 days), as is the case in the Shift and Truncate (SANT) model, censoring is necessary to prevent implausible future dates that result in truncated data availability and increase the risk of re-identification, thereby weakening the shifting algorithm.^37^ Similarly, datasets that suppress patient populations, such as newborns or elderly individuals, limit the relevance of longitudinal analyses.

Data curators seek to expand the breadth and relevance of the dataset by record linkage across various resources that provides a more comprehensive view of a patient's experience by matching records for the same individual, entity, or event in a privacy-preserving manner. Over the past decade, tracking techniques, such as probabilistic matching and tokenization (instead of shared identifiable data), have become powerful tools for integrating new data elements (eg, SDOH) and improving completeness (eg, mortality records).^38^ The impact on RWE is improved confounding control and outcome identification, resulting in more accurate and reliable results.^45^ This process is limited to datasets with shared tokens and may introduce selection bias and reduce generalizability.^46^ Additionally, when a tokenized dataset uses date shifting, linkage is only attainable if identical date-shifting algorithms are applied, typically by a third party (honest broker) to reduce the re-identification risk.^47^ Most other de-identification techniques do not affect the ability to link RWD across sources, apart from synthetic data, where linkage is irrelevant since patients are fictitious. Importantly, linkage of 2 de-identified datasets that use different de-identification methodologies must undergo and document an additional compliance or legal review as the relationships between quasi-identifiers and the population characteristics may have shifted.

The curation of relevant and reliable data depends on data availability, which refers to the extent to which data are present, complete, and accessible for the intended goals.^5-8,39^ Data availability is both affected by and encompasses most other concepts in the scope of relevance and reliability, such that available data improve data accuracy and increase data granularity. De-identification techniques may remove entire fields or specific codes within a field using tracking, masking with substitution or blurring, and redaction via filtering or suppression; this removal impacts the relevance of the dataset. For instance, de-identification may remove by suppression COVID-19–related diagnostic, procedure, and care location codes since they increase a patient's risk of re-identification due to the public reporting of COVID-19 cases during the epidemic and produce underestimated rates of outcomes and care during that time period.^48^ Alternately, the complete redaction of a patient(s) records is possible in cases where the data represent extreme outliers with the potential to impair availability of the data.^49^ Documentation of the rationale for the removal of outlier patients is a necessary quality step to understand potential biases.^50^

The accuracy of the fit-for-purpose real-world EHR dataset is the true reflection of the patient and their associated health care data.^5-7,39^ De-identification techniques that alter or remove these data, such as tracking, masking, redaction, disruption, and randomization, reduce the ability to validate assertions based on the original dataset. De-identification techniques of partial redaction and generalization require proper lineage to understand the potential for misclassification bias. For instance, generalization can increase the impact of misclassification bias as in the case of aggregating race into several large categories and the additional response options, such as “refuse to answer” or “not applicable” into either “other” or “missing.”^51^ Top coding is another case of reduced data accuracy, where, for example, patients older than 90 years are recoded as 89 years. For the de-identification technique of date shifting that alters source values, the preserved granularity (see below) of relative daily changes is essential for understanding research such as pre- and postsurgical care management, vaccine safety, and medication switching.^52^ When date shifting does not account for care related to day of the week or season, data relevance is reduced. In the example from the Centers for Disease Control and Prevention Wide-ranging Online Data for Epidemiologic Research (CDC WONDER), data with fewer than 10 case counts were suppressed and the resultant mortality rates and disease burden in rural areas were consistently underestimated.^53,54^ Data randomization strives to closely match the actual measured values across the patient population whereby data performance mimics source data for training predictive models, developing decision support for health care professionals, and designing synthetic controls for clinical trials.^55^ For subsampling, no changes are imposed on the source data; however, the reliability of the data may be limited by incomplete distributions across all potential categories or values.

Finally, data granularity is the detailed values available to define and characterize a field and often critical to the intended research. De-identification techniques applied to reduce variable granularity lower the distinguishability of a patient's data. Masking techniques, generalization, disruption, and randomization using subsampling are all techniques that, assuming proper documentation of transformations (ie, lineage), do not eliminate data relevance, although reduce data accuracy. For example, the granularity of a patient's geography of residence zip code (eg, the first 3 or 5 digits) may improve the understanding disparities in health care burden, access, and inclusion in interventions or clinical trials.^56,57^ Generalization of sexual orientation and gender identity data, which are increasingly being integrated in EHR systems, into broad categories of female, male, and other/missing have been shown to increase biases in health care.^58^

## Discussion

Data utility requirements for privacy-compliant RWD datasets are not explicitly regulated in the United States or Europe. Fundamentally, patient privacy laws ensure data availability but do not account for their usability. It is the data curators who balance privacy protection with the preserving of data utility—defined broadly as availability, relevance, and reliability of information.^9,20^

Beyond a generalized compliance attestation, we advocate for transparency and methodological rigor in privacy practices, recognizing the growing need for such approaches and adoption.^25,59-62^ Pilgram et al,^59^ for example, examined the impact of data anonymization techniques using data utility metrics for granularity, entropy, and reproducibility. Similarly, Lee and You^60^ described the impact of data anonymization on the accuracy of computer vision. Last, the Tranforming Real-World Evidence with Unstructured and Structured Data to Advance Tailored Therapy (TRUST) study provided novel tools to assess the accuracy, completeness, and traceability of RWD studies.^61^

Ideally, de-identification and anonymization practices are reported and disaggregated into their constituent techniques so that important insights can be gained regarding potential limitations and biases that affect the dataset's utility. For example, tokenization increases data completeness by allowing for record linkage, while date shifting improves data relevance and generalizability by reducing re-identification risks and enabling the retention of quasi-identifier details, such as for racial and ethnic characteristics. Additionally, researchers may need to report on layers of curation as data go from health care providers to third-party aggregators, such as for registries. In certain use cases, for example, datasets may be subject to state-specific legislation, funding sources, or vendor-driven practices, which may include an additional application of privacy-enhancing technologies safeguarding against re-identification.^14,63^ It is important to recognize that compliance and conformance of RWD for RWE extend beyond EHR data, which also require careful curation and transparency with documentation. These increasingly diverse sources of patient data include non–HIPAA-protected health-related data, such as patient-generated data, direct-to-consumer genetic testing, and geolocation data that can be directly integrated into clinical care or linked in postprocessing steps. Finally, while privacy safeguards focus on identity protection, they may not address the consequences of how data inferences affect individuals or groups broadly or how those data are ultimately applied.

## Conclusion

Health care professionals, researchers, policymakers, and regulators increasingly recognize the abundance and potential of RWD, with a growing emphasis on ensuring that the RWE derived from such data is generated from privacy-compliant, methodologically sound, and high-quality RWD. Real-world data originating from EHRs involve a complex set of trade-offs beginning with patient data accrual across disparate networks and platforms, through documentation by a heterogenous population of health care providers and systems and culminating into standardized and normalized data formatted within an interoperable common data model.

As outlined herein, the curation of these data into reliable and relevant real-world fit-for-purpose data requires adherence to and careful consideration of the operational impact of privacy laws and regulations. Central to this is the comprehensive documentation of de-identification techniques used during data management that is foundational to achieving privacy compliance. Such documentation should explicitly account for the data's intended use, accessibility, timeliness, linkage, availability, accuracy, and granularity, all within the context of the de-identification methods used. A more transparent process will lead to more informed, effective, and ideally, more valid insights by researchers and decision-making by policymakers.

## Supplementary Material

qxaf210_Supplementary_Data
