# Supplementary material for: A real-world data challenge: guidance for aligning data privacy compliance and fit-for-purpose usability
Source: Health Aff Sch. 2025 Nov 12;3(11):qxaf210. doi: 10.1093/haschl/qxaf210 (PMC12661526; doi:10.1093/haschl/qxaf210)
Supplement: qxaf210_Supplementary_Data [file qxaf210_supplementary_data.zip › qxaf210_Supplementary_Data.pdf]

Appendix

Table S1. Guidance for management and utilization of electronic health records for real-world evidence

RWD Management		Utilization for RWD Study	
ISPOR SUITABILITY Framework[a]	FDA Fit-for-Purpose Assessment[b]	ISPOR SUITABILITY Framework[a]	FDA Fit-for-Purpose Assessment[b]
Characteristics<br>• Meta data (platform, version, accrual and curation, CDM and mappings, validation rules, data types, coverage, timeliness)<br>• Dataset purpose<br>• Linkage<br><br>Provenance<br>• Traceability<br>• Transformations and resultant quality as part of privacy and data usability<br><br>Governance<br>• Governance body<br>• Regulatory adherence to privacy standards<br>• Data access<br>• Data funding source	Data reliability<br>• Accrual<br>• Quality and integrity (including traceability and provenance)<br><br>Design relevance<br>• Data availability<br>• Linkage<br>• Timeliness	Reliability<br>• Completeness<br>• Accuracy<br><br>Relevance<br>• Content<br>• Setting and timing<br>• Sample size and follow-up	Data reliability<br>• Completeness<br>• Accuracy<br><br>Design relevance<br>• Study purpose<br>• Data elements<br>• Generalizability<br>• Confounding assessment<br>• Timing of data

[a] Adapted from Fleurence et al.[1]
[b] Adapted from US FDA 2023.[2]
Abbreviations: CDM, common data model; FDA, Food and Drug Administration; ISPOR, International Society for Pharmacoeconomics and Outcomes Research; RWD, real-world data

Notes:

1. Fleurence RL, Kent S, Adamson B, et al. Assessing Real-World Data from Electronic Health Records for Health Technology Assessment: The SUITABILITY Checklist: A Good Practices Report of an ISPOR Task Force. *Value Health*. 2024;27(6):692-701. doi:10.1016/j.jval.2024.01.019
2. US Food and Drug Administration. *Use of Real-World Evidence to Support Regulatory Decision-Making for Medical Devices Draft Guidance for Industry and Food and Drug Administration Staff Draft Guidance*. US DHHS; 2023. https://www.fda.gov/media/174819/download
